# Author Correction: Data-driven quantum chemical property prediction leveraging 3D conformations with Uni-Mol+

**DOI:** 10.1038/s41467-024-52804-6

**Published:** 2024-10-02

**Authors:** Shuqi Lu, Zhifeng Gao, Di He, Linfeng Zhang, Guolin Ke

**Affiliations:** 1 DP Technology, Beijing, China; 2 Peking University, Beijing, China

**Keywords:** Computational science, Computational chemistry

Correction to: *Nature Communications* 10.1038/s41467-024-51321-w, published online 19 August 2024

In this article, the affiliation details for Zhifeng Gao were incorrectly given as ‘Peking University, Beijing, China’ but should have been ‘DP Technology, Beijing, China’. Second, the affiliation details for Di He were incorrectly given as ‘DP Technology, Beijing, China’ but should have been ‘Peking University, Beijing, China.’ The original article has been corrected.

