# Supplementary material for: Data-driven quantum chemical property prediction leveraging 3D conformations with Uni-Mol+
Source: Nat Commun. 2024 Aug 19;15:7104. doi: 10.1038/s41467-024-51321-w (PMC11333583; doi:10.1038/s41467-024-51321-w)
Supplement: Supplementary file 1 — Supplementary Information [file 41467_2024_51321_MOESM1_ESM.pdf]

Supplementary Information for: Data-driven Quantum Chemical Property Prediction Leveraging 3D Conformations with Uni-Mol+

Shuqi Lu[1], Zhifeng Gao[1], Di He[2], Linfeng Zhang[1], Guolin Ke[1*]

[1]DP Technology, Beijing, China.
[2]Peking University, Beijing, China.

*Corresponding author(s). E-mail(s): kegl@dp.tech;
Contributing authors: lusq@dp.tech; gaozf@dp.tech; dihe@pku.edu.cn; zhanglf@dp.tech;

1 More Discussion of Model Backbone

Comparison with Evoformer of AlphaFold2

`Uni-Mol+` shares similarities with AlphaFold2 [1]'s Evoformer, especially in how both use a two-track transformer approach (Node Representation and Pair Representation). However, the key difference lies in the type of input data each one uses. Evoformer is built to process protein sequences and multiple sequence alignments (MSAs), whereas `Uni-Mol+` is designed for atoms and their 3D positions. This fundamental difference leads to several variations in their design.

For instance, Evoformer includes specific layers like MSARowAttention and MSAColumnAttention to handle MSAs, which are not needed in `Uni-Mol+`. In the Pair Representation, `Uni-Mol+` uses a single TriangularUpdate for efficiency, while Evoformer uses four different Triangular operators. Furthermore, Evoformer relies on a complex StructureModule to predict the protein structure from sequence representation, while `Uni-Mol+` simply needs a SE(3) coordinate head. As a result, `Uni-Mol+` is simpler and more efficient compared to Evoformer.

2 More Experiments

Robustness regarding initial conformations

We have conducted additional experiments to assess the robustness of our model with varying input conformations. Specifically, we introduced Gaussian noise (with standard deviations of 0.1, 0.2, and 0.3) to the initial RDKit conformations. Besides, we explored the effects of employing conformations obtained through random perturbations of torsion angles by introducing Gaussian noise with standard deviations of 0.005π, 0.01π and 0.02π to every torsion angle in the conformations generated by RDKit. The results, as detailed in Supplementary Table 1, demonstrate that our model's performance is relatively unaffected by changes in the initial conformations.

Furthermore, we conducted an experiment starting from 2D conformations (with a flat z-axis) generated by RDKit's `AllChem.Compute2DCoords`. Despite the significant challenge posed by the absence of 3D information, the result is only a minor drop in performance and still largely outperform previous baselines. This finding underscores the robustness of `Uni-Mol+`: it maintains high performance levels even without 3D conformation inputs.

Supplementary Table 1 The benchmark results on PCQM4MV2, with different initial conformations. Source data are provided as a Source Data file

Method	Valid MAE (↓)
`Uni-Mol+`	0.0695
`Uni-Mol+` w/ Noisy RDKit Conf., std=0.1	0.0695
`Uni-Mol+` w/ Noisy RDKit Conf., std=0.3	0.0694
`Uni-Mol+` w/ Torsion Angle Perturbed RDKit Conf., std=0.005π	0.0698
`Uni-Mol+` w/ Torsion Angle Perturbed RDKit Conf., std=0.01π	0.0695
`Uni-Mol+` w/ Torsion Angle Perturbed RDKit Conf., std=0.02π	0.0696
`Uni-Mol+` w/ 2D Conf.	0.0715

Time Cost Regarding Molecular Sizes

To analyze the time cost of `Uni-Mol+`, we chose a diverse set of molecules, grouping them by size, and calculated the average time required to process a single molecule in each category, including 50 molecules per size group.

We compared the time costs between `Uni-Mol+` across different numbers of conformation update rounds (R) and traditional Density Functional Theory (DFT) calculations. Specifically, for `Uni-Mol+`, we assessed the computational time for molecules with up to 256 atoms. However, due to the substantial time demands of DFT calculations, we limited our DFT time cost analysis to molecules with a maximum of 50 atoms.

The computational evaluations for `Uni-Mol+` were performed on a single NVIDIA V100 GPU, while the DFT calculations, including geometry optimization and quantum chemical energy computations, were conducted using Psi4 1.4.1 on 32 CPUs. The DFT calculations employed the B3LYP functional and 6-31G* basis set, aligning with the settings used in the PCQM4MV2 dataset.

We have presented these results in Supplementary Fig. 1. The results clearly indicate that `Uni-Mol+` not only provides a faster computational solution compared to DFT but also demonstrates superior scalability in relation to molecular size. Moreover, our findings reveal that the computational time cost associated with `Uni-Mol+` increases linearly with the number of update rounds (R), affirming the predictability and efficiency of our method.

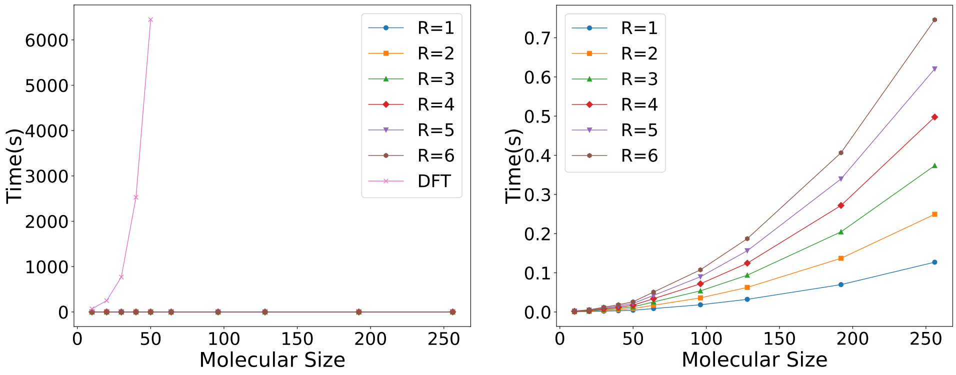

(a) (b)

Supplementary Fig. 1 Time cost for different molecular sizes. (a) DFT versus `Uni-Mol+` (with varying update rounds R); (b) Effect of different update rounds R in `Uni-Mol+`. Source data are provided as a Source Data file

Valid MAE Regarding Molecular Sizes

We've extended our analysis to explore how conformation update rounds (R) and molecule sizes influence error metrics, presented in Figure 2 and Table 2. This analysis revealed a notable trend: larger molecules tend to incur higher errors compared to smaller molecules. Interestingly, our data indicates that increasing "R" does not significantly benefit larger molecules, as the performance at R=1 and R=2 is nearly identical.

Upon further examination, we recognized that the amount of larger molecules is quite small in validation set, as shown in Table 2. This led us to investigate the training data's molecular size distribution, detailed in Table 3. Most molecules in the training set are within the size range of (10, 20], which correlates with where we observe the lowest validation MAE. This indicates the the higher errors on larger molecules is due to the distribution of training data.

Quantitative Analysis for the Optimized Conformations

We extended quantitative analysis for the optimized conformations to the OC20 benchmark, where ground-truth equilibrium conformations are accessible within the validation dataset. We have conducted additional evaluations and benchmarked our results against the current state-of-the-art model, EquiFormer [2]. The results, as

Supplementary Table 2 The prediction error of increasing R on molecules grouped by different sizes. Source data are provided as a Source Data file

molecular size	num	R	Valid MAE (↓)
10	10404	1	0.0767
		2	0.0787
20	59865	1	0.0664
		2	0.0660
30	3146	1	0.1097
		2	0.1096
40	106	1	0.1622
		2	0.1543
50	23	1	0.1769
		2	0.1789

Supplementary Table 3 Data distribution of Training set. Source data are provided as a Source Data file

molecular size	num
10	319135
20	3059203

depicted in Supplementary Table 4, clearly demonstrate that `Uni-Mol+` surpasses the previous baseline in predicting equilibrium conformations.

Supplementary Table 4 RMSD for predicted conformations on OC20 valid set. Source data are provided as a Source Data file

	RMSD↓			
Model	ID	OOD Ads.	OOD Cat.	OOD Both
EquiFormer	1.7622	1.7277	1.0157	1.8116
`Uni-Mol+`	**1.5067**	**1.4756**	**0.9281**	**1.5401**

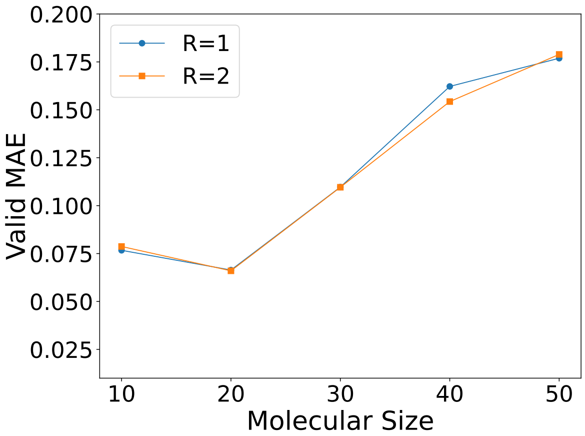

Supplementary Fig. 2 The prediction error of increasing R on molecules grouped by different sizes. Source data are provided as a Source Data file

References

[1] John Jumper, Richard Evans, Alexander Pritzel, Tim Green, Michael Figurnov, Olaf Ronneberger, Kathryn Tunyasuvunakool, Russ Bates, Augustin Žídek, Anna Potapenko, et al. Highly accurate protein structure prediction with alphafold. *Nature*, 596(7873):583–589, 2021.

[2] Yi-Lun Liao and Tess Smidt. Equiformer: Equivariant graph attention transformer for 3d atomistic graphs. *arXiv preprint arXiv:2206.11990*, 2022.
