# Supplementary material for: Data-driven quantum chemical property prediction leveraging 3D conformations with Uni-Mol+
Source: Nat Commun. 2024 Aug 19;15:7104. doi: 10.1038/s41467-024-51321-w (PMC11333583; doi:10.1038/s41467-024-51321-w)
Supplement: Supplementary file 3 — Description of Additional Supplementary Files [file 41467_2024_51321_MOESM3_ESM.pdf]

Description of Additional Supplementary Files

Supplementary Data 1

1. Overview of the Supplementary Data:
The supplementary data provided includes detailed conformation structures shown in Figure 2 and Figure 3 in the main manuscript.

2. File Descriptions:
   (1) conformation_compare_fig2: contains the initial conformations, predicted conformations, and DFT conformations shown in Figure 2.
   (2) conformation_energy_fig3: contains the initial conformations and the model-predicted conformations of the the data points in Figure 3.

3. Data File Details:
   (1) conformation_compare_fig2: files with the suffix '_init_align.sdf' are the initial conformations, '_target_align.sdf' are the DFT conformations, '_0_align.sdf' are the predicted conformations at R=0, and '_1_align.sdf' are the predicted conformations at R=1.
   (2) conformation_energy_fig3: files with the suffix '_init.sdf' are the initial conformations and '_1.sdf' are the predicted conformations.
